# Supplementary material for: The interactome of intact mitochondria by cross-linking mass spectrometry provides evidence for coexisting respiratory supercomplexes
Source: Mol Cell Proteomics. 2017 Dec 8;17(2):216–32. doi: 10.1074/mcp.RA117.000470 (PMC5795388; doi:10.1074/mcp.RA117.000470)
Supplement: Supplemental Data [file supp_RA117.000470_133922_0_supp_23978_fzffwf.zip › spectra_annotation/mito_spectra_annotation/55-1-17-1-4-1.pdf]

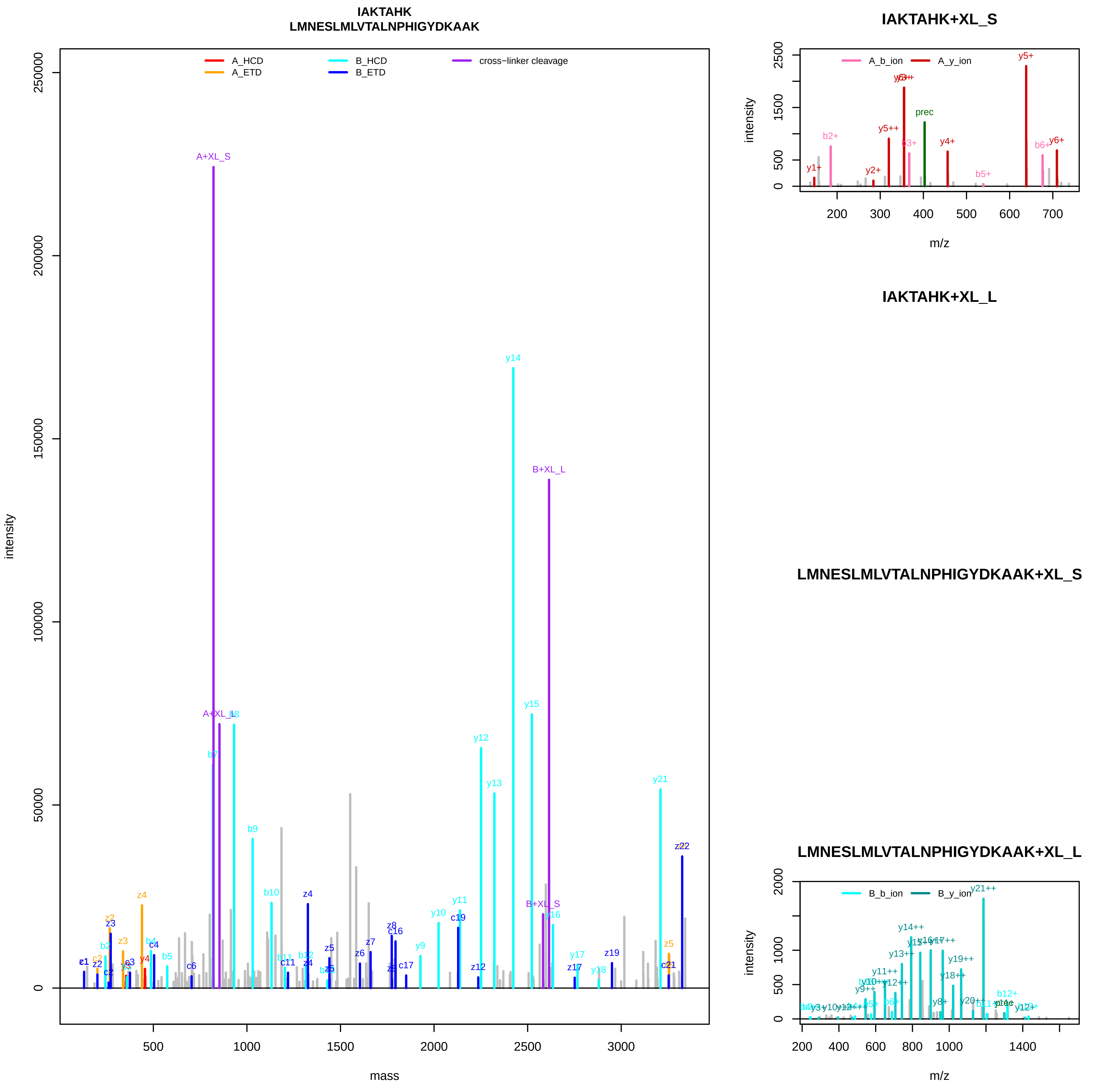
IAKTAHK
LMNESLMLVTALNPHIGYDKAAK
A_HCD
A_ETD
B_HCD
B_ETD
cross−linker cleavage
A+XL_S
A+XL_L
B+XL_L
B+XL_S
intensity
mass
0
50000
100000
150000
200000
250000
500
1000
1500
2000
2500
3000
y14
y15
y12
y13
y21
y16
y17
y18
y9
y10
y11
b7
b9
b10
b11
b12
b5
b4
b2
b3
c1
c2
c3
c4
c6
c11
c16
c17
c19
c21
z1
z2
z3
z4
z5
z6
z7
z8
z12
z17
z19
z22
y4
IAKTAHK+XL_S
A_b_ion
A_y_ion
y1+
y2+
y4+
y5+
y5++
y6+
b2+
b3+
b5+
b6+
prec
m/z
IAKTAHK+XL_L
LMNESLMLVTALNPHIGYDKAAK+XL_S
LMNESLMLVTALNPHIGYDKAAK+XL_L
B_b_ion
B_y_ion
y21++
y14++
y13++
y19++
y18++
y11++
y12++
y9++
y8+
y20++
b12+
prec
200
400
600
800
1000
1400
